# Research Progress on Skin Aging and Active Ingredients

**DOI:** 10.3390/molecules28145556

**Published:** 2023-07-20

**Authors:** Xin He, Fang Wan, Wenhui Su, Weidong Xie

**Affiliations:** 1 State Key Laboratory of Chemical Oncogenomics, Shenzhen International Graduate School, Tsinghua University, Shenzhen 518055, China; hexin22@mails.tsinghua.edu.cn (X.H.); wanf21@mails.tsinghua.edu.cn (F.W.); suwh22@mails.tsinghua.edu.cn (W.S.); 2 Open FIESTA Center, Shenzhen International Graduate School, Tsinghua University, Shenzhen 518055, China; 3 Shenzhen Key Laboratory of Health Science and Technology, Institute of Biopharmaceutical and Health, Tsinghua University, Shenzhen 518055, China

**Keywords:** anti-aging ingredients, inflammation, photoaging, reactive oxygen species, skin aging

## Abstract

With the advancement of living standards in modern society and the emergence of an aging population, an increasing number of people are becoming interested in the topic of aging and anti-aging. An important feature of aging is skin aging, and women are particularly concerned about skin aging. In the field of cosmetics, the market share of anti-aging products is increasing year by year. This article reviews the research and development progress of skin aging and related active compounds both domestically and internationally in recent years. The results show that, in terms of the research on skin aging, the popular theories mainly include free radicals and oxidative stress theory, inflammation theory, photoaging theory, and nonenzymatic glycosyl chemistry theory. In terms of research on the active ingredients with anti-aging activities in the skin, there are numerous reports on related products in clinical studies on human subjects, animal experiments, and experimental studies on cell cultures, with a variety of types. Most of the compounds against skin aging are sourced from natural products and their action mechanisms are mainly related to scavenging oxygen free radicals and enhancing antioxidant defenses. This review provides important references for the future research of skin aging and the development of related products. Although there is a great progress in skin aging including related active ingredients, ideal compounds or products are still lacking and need to be further validated. New mechanisms of skin aging, new active ingredients sourced from natural and artificial products, and new pharmaceutical forms including further clinical validations should be further investigated in the future.

## 1. Introduction

Currently, the global population will continue to experience aging, with some developed and developing countries in particular moving towards severe aging populations in their societies. South Korea and Japan will become the two countries with the most severe aging populations [1]. Some experts point out that China will exhibit a moderately-aging society in 2023. According to a report, in 2022, the population aged 60 and above will have reached 28.04 million, accounting for 19.8%; among them, the population aged 65 and above was 209.78 million, accounting for 14.9% [2]. As the population is becoming an aging society and the living standards are improving, there is now a growing interest in the topic of aging among modern societies as they seek to understand and counter the adverse effects of aging. One of the most prominent features of aging is skin aging, which is of particular concern to women. According to the statistics, the proportion of Chinese women taking anti-aging measures is 90%, and the size of China’s anti-aging market reached CNY 64.6 billion in 2020 [3].

The main characteristics of skin aging are skin relaxation and wrinkles [4], which are related to skin cell aging and decreased collagen synthesis or increased degradation [5,6]. The causes of skin aging can be divided into endogenous and exogenous factors [7]. Endogenous factors mainly involve the accumulation of time and the influence of related physiological traits, while exogenous factors mainly include ultraviolet radiation, smoking, wind exposure, and exposure to harmful chemicals [8,9]. These incentives, especially exogenous ones, will cause macromolecular structural damage and related functional changes in skin cells, thus leading to accelerated skin aging [10]; however, the mechanisms of skin aging and effective compounds or products against skin aging are not well understood.

New compounds or drugs to be used against skin aging are increasingly added into books and onto shelves. The current compounds or drugs used against skin aging can be orally administrated or locally applied onto the skin according to their characteristics of pharmacokinetics; however, for oral administration, a low bioavailability could occur and it may be difficult for those compounds to reach the skin tissues. For local application on the skin, these compounds or drugs still have limitations in their chemical stability and a lack of transdermal absorption, limiting their application in the cosmetics field. These compounds or drugs, therefore, should be further improved in pharmaceutical preparations, and a new drug delivery system may enable these chemicals to have a better absorption and better skin anti-aging activity [11].

With the development of biology, medicine, chemistry and pharmaceutics, the field of skin aging and active ingredients has made great progress. Here, we attempt to systematically review the main mechanisms of skin aging and effective compounds or products for use against skin aging.

## 2. Mechanisms of Skin Aging Processes

At present, classic theories on the mechanism of skin aging include the theory of free radicals and oxidative stress, the theory of inflammatory aging, the theory of skin photoaging, and the theory of nonenzymatic glycosyl chemistry. These main theories are briefly described below.

⮚ Free radicals and oxidative stress theory

Free radicals are one of the main causes of decreased body function and skin aging. Reactive oxygen species (ROS) are a type of unstable molecule that contain oxygen and that easily react with other molecules in a cell. Oxidative stress refers to the imbalance between intracellular oxidation and antioxidant activity, where cells tend to oxidize and produce a large amount of ROS. During cell metabolism, mitochondria produce ROS through oxidative metabolism. When there is too much ROS in cells, the mitochondria will be damaged, the production of mitochondrial ATP will be reduced, the mitochondrial membrane potential will be reduced, and a chain reaction will be generated to accelerate aging [12]. Excessive ROS can also damage the DNA structure, causing aging symptoms such as cell function damage and cell replication disorders [13,14]. A significant increase in the ROS levels not only accelerates skin replication aging, but also promotes a decrease in the collagen levels in skin tissue, leading to skin relaxation and wrinkles [5]. The molecular mechanism is related to the increase in matrix metallopeptidase (MMP) expression [15,16,17,18]. MMP is a kind of zinc-dependent endogenous protease. MMP can specifically degrade the extracellular matrix, including collagen, and can cause damage to the extracellular matrix, which ultimately leads to skin aging. Consequently, removing excess ROS from the skin cells has become one of the most common ways to combat skin aging [15].

⮚ Inflammation theory

Inflammation is one of the major causes of cellular senescence. “Inflammatory aging” is characterized by increased levels in the proinflammatory factors in the body. These changes will lead to the aging of body cells, including the skin, and induce many aging diseases [19,20]. At the skin level, senescent fibroblasts and keratinocytes secrete a large number of “senescence associated secretory phenotypes (SASP)”, including the proinflammatory cytokine TNF-α, IL-1, IL-6, IFN-γ and MMPs and others [21]. These pro-inflammatory cytokines induce skin cell senescence by promoting the production of ROS and activating the ATM (ataxia telangiectasia mutated)/p53/p21-signaling pathway. At the same time, when skin cells develop inflammation, this will lead to an increased release of MMPs, which will cause the degradation of collagen, resulting in the relaxation of and wrinkles in the skin cells [22,23]. Inhibiting skin cell inflammation is, therefore, one of the important strategies to control skin cell aging.

⮚ Photoaging theory

External factors such as ultraviolet (UV) light in sunlight play a very important role in the process of skin aging. UV plays a diverse role in the anti-aging of skin, with different wavelengths having diverse effects and systemic implications. The specific wavelength of UVR determines the nature of the signals it transduces, affecting the local neuroendocrine axes, and may induce skin aging [24]. According to the mechanism theory of photoaging, ultraviolet light can lead to the production of ROS and the secretion of MMPs [25]. Long term exposure to solar ultraviolet radiation will cause photoaging, which will affect pigmentation, immunity and the vascular system [26,27]. Adult dermal collagen content decreases every year, and the decrease in collagen is mainly caused by the increase in MMP expression and the decrease in collagen synthesis [28]. With an increase in age, the levels of MMP-1, 2, 9 and 12 increase, while the expression of procollagen mRNA decreases significantly, which leads to a decrease in dermal collagen content. Significant evidence has proved that MMP plays a major role in inducing the onset of photoaging. UV irradiation induces keratinocytes and fibroblasts to secrete MMPs, which in turn degrade the dermal extracellular matrix components such as collagen [29]. Inhibiting skin UV irradiation and its related damage is one of the important strategies to prevent skin cell aging.

⮚ Nonenzymatic glycosyl chemistry theory

Internal factors such as nonenzymatic glycosylation (known as a Maillard reaction) also play a very important role in skin cell aging. According to this theory, the crosslinking damage of proteins caused by glycosylation is the main reason for aging [30]. This glycosylation is a nonenzymatic reaction between free reducing sugars and free amino groups of proteins, DNA and lipids, resulting in advanced glycation end products (AGEs) [31]. The accumulation of AGEs will affect cellular homeostasis and protein structure changes, leading to skin darkening and aging. The accumulation of AGEs also leads to ROS production and inflammation, thus accelerating skin aging, and the formation of AGEs is irreversible. Moreover, skin cells age at high levels of glycation [32,33,34]. With an aging of the population, the number of diabetic patients will increase significantly, and skin glycosylation will be more common [35]. Inhibiting skin glycosylation is also one of the important ways to control skin aging.

In addition to the four theories mentioned in this article, genetical factors, DNA repair and stability, cellular senescence and telomeres, mitochondria, apoptosis, estrogen deficiency, circadian rhythms, neuroendocrine, diseases, physical activity, stress, and other environmental factors (e.g., diet, pollution and smoking) are also important in the field of skin aging, and have been studied in animal and experimental studies on cell cultures [36,37,38,39,40]. Additionally, skin as a stress sensor, modulates homeostasis via the neuroendocrine and immune systems, and signaling molecules. It protects against stressors through keratinocyte differentiation, the pigmentary system, and communication with the dermis/hypodermis. Dysfunctions can cause various skin disorders and aging [41].

Based on the descriptions above, the mechanisms of skin aging are mainly related to an increase in the intracellular ROS level and oxidative stress, an increase in the inflammatory level, and a subsequent decrease in the collagen level. Excessive external UV irradiation and an enhanced internal microenvironment such as glycation are also important factors that lead to skin aging, but the basic mechanism is still related to the upregulation of skin cell ROS and an increase in oxidative stress and inflammation. It is, therefore, very critical to inhibit the production of skin cell ROS in order to attenuate the aging of the skin. In addition, the direct upregulation of collagen level is also one of the important strategies to repair skin cells and reduce skin wrinkles. 

**Figure 1:**
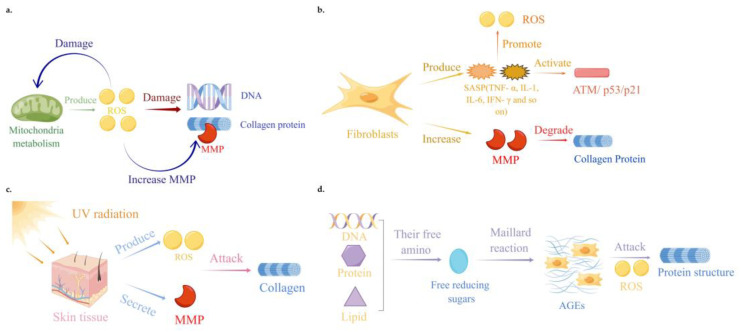
Mechanisms of skin aging processes. (**a**) Free radicals and oxidative stress theory. Mitochondria produce ROS through oxidative metabolism. Excessive ROS can damage the mitochondrial and DNA structures, leading to a decrease in collagen levels and an increase in MMP levels in skin tissue. (**b**) Inflammation theory. Senescent fibroblasts and keratinocytes secrete a large number of senescence-associated secretory phenotypes, including TNF-α, IL-1, IL-6, IFN-γ and MMPs. These proinflammatory cytokines induce skin cell senescence by promoting ROS production and activating the ATM/ p53/p21-signaling pathway. (**c**) Photoaging theory. Ultraviolet irradiation induces the production of ROS and the secretion of MMPs, which degrades skin extracellular matrix components such as collagen. (**d**) Nonenzymatic glycosyl chemistry theory. Non-enzymatic glycosylation is a reaction between free reducing sugars and free amino groups of proteins, DNA and lipids to produce AGEs and ROS. The accumulation of AGEs, together with ROS, can lead to changes in the cell homeostasis and protein structure. These images were drawn with the Figdraw software1.0.

A summary of the mechanism of the skin aging processes is shown in Figure 1.

## 3. Research on Skin Anti-Aging Compounds

In recent years, skin anti- aging products have developed rapidly. In the following, the anti-aging compounds and their related products are presented from the perspective of the type of published data (e.g., on humans, on animals and on cell cultures) on which their development as compounds for anti-aging products has been based.

◆ Data from clinical studies on human subjects:
  ● Ceramide: Ceramide has a protective effect on the skin from external pressure, which can prevent skin dryness and play a role in the skin barrier [42]. A group of 30 healthy adults (including women and men) aged 20 to 60 years old were selected and they consumed dietary supplements (including 1197 mg of acetic acid bacteria containing 9.06 mg of ceramide) for four consecutive weeks. Physical measurements, blood tests, and urine analyses showed that ceramide can preserve the skin barrier [43]. This reveals that ceramide has a role in protecting the skin and resisting the influence of external substances on skin aging.
  ● Retinol: Retinol can significantly improve the microenvironment of dermal ECM, stimulate skin cells to produce ECM (e.g., type I collagen, fibronectin and elastin), can promote the formation of dermal blood vessels, and can stimulate TGF-β/CTGF, which is the main regulatory factor for maintaining an ECM steady-state [44]. Women (n = 24) aged 18–65 with acne, pigmentation, or melasma were selected and they applied a 3% retinol for six consecutive weeks. The results showed that the skin of the subjects showed significant improvements in fine lines, wrinkles, pore size, relaxation, pigmentation spots, clarity, brightness, and overall light damage [45]. This indicates that 3% retinol is an effective drug for treating skin aging.
  ● Hyaluronic acid: The most obvious clinical feature of photoaging is the appearance of wrinkles, and the tissue feature is the accumulation of abnormal elastin in the dermis and a serious loss of collagen fibers. The integrity of elastin and collagen fibers in the dermis is maintained by their interaction with hyaluronic acid and a proteoglycan network structure; therefore, hyaluronic acid degradation may be the initial process before the decomposition of fibril components [46]. Female subjects (n = 60) with skin aging were selected to participate in clinical trials, taking 200 mg of hyaluronic acid orally, daily, for 28 consecutive days. After 28 days, the skin wrinkles and moisture content of the subjects were tested, and the results showed that hyaluronic acid can increase the skin moisture content, reduce the wrinkle depth, and increase the skin elasticity and strength [47]. This reveals that hyaluronic acid has the effect of improving skin aging.
  ● Astaxanthin: Astaxanthin has antioxidant and anti-inflammatory effects, and it can reduce skin photoaging [48]. Astaxanthin is related to a variety of anti-inflammatory mechanisms, including PI3k/Akt, Nrf2, NF-κB. Erk1/2, JNK, p38 MAPK and jak-2/stat-3 [49]. Healthy female subjects (n = 23) were selected to take orally astaxanthin capsules containing 4 mg for 10 weeks. The skin erythema dose, skin water content and other parameters of the subjects were measured. The results showed that the astaxanthin-administration group had a reduced skin moisture loss, an improved skin roughness, and that skin degradation caused by UV light was prevented [50]. This revealed that astaxanthin has the effect of improving skin aging.
  ● Collagen tripeptide (Gly-Pro-Hyp): Collagen derived tripeptide (Gly Pro Hyp) can prevent a reduction in the extracellular matrix (ECM)-related genes COL1A, elastin and fibronectin [51]. Female subjects (n = 64) were randomly and double-blind selected, to take 1000 mg of Gly-Pro-Hyp orally once a day for 12 weeks. After 12 weeks, the skin moisture content, wrinkles, and elasticity of the subjects were tested. The results showed that compared with the control group, the skin moisture content of the subjects in the treatment group increased, wrinkles decreased, and elasticity improved [52]. This reveals the role of Gly-Pro-Hyp in skin anti-aging.
  ● Acetylzinc ketone (AZ): Acetylzinc ketone (AZ), a derivative of zinone and a phytochemical in ginger, is a new compound with a photoaging effect. AZ can increase the expression of the Notch pathway genes, reduce MMP-1, MMP-3, and MMP-12, and can also reduce the expression of the reactive oxygen species-related genes PMAIP1 and arg2 [53]. Healthy female participants (n = 31, aged 44 ± 7 years) were randomly selected and smeared twice a day for 8 weeks. Facial image analysis photography and software were used to evaluate the signs of photoaging, including wrinkles, pigmentation and redness. The results showed that compared with the control group, the average wrinkle severity, total wrinkle volume, pigment intensity and redness intensity of subjects in the administration group were significantly reduced within 8 weeks [54]. This indicates that AZ plays a significant role in resisting skin aging.
  ● Epigallocatechin Gallate (EGCG): EGCG has strong antioxidant activity and can significantly reduce intracellular reactive oxygen species [55]. Female healthy subjects (n = 88) were selected and supplied with Hanhoo cosmetics (i.e., Hanhoo organic tea stamen whitening water, with 30 mL of Hanhoo organic tea stamen extremely tender essence, and Hanhoo organic tea stamen tender white emulsion or whitening cream) containing epigallocatechin gallate EGCG for 12 weeks, which was applied by smearing the skin of the subjects. The results showed that the EGCG components could have scavenged ROS in the subjects, thus preventing DNA damage caused by UV light [56], with the effect of resisting skin aging.
  ● Vitamin C: Vitamin C is a nutrient necessary for a variety of biological functions. It is a scavenger of reactive oxygen species [57] and has the effect of preventing lipid peroxidation. Using a cosmetic containing 20% vitamin C, 50 randomly-selected female subjects were tested. The vitamin C was applied to their face every day. The skin melanin index, elasticity, gloss, moisture, smoothness, roughness, scaliness and wrinkles were detected at the 4th and 8th weeks. The results showed that vitamin C could improve the color, elasticity and luster of the skin [58]. This suggests that vitamin C has a potential anti-skin aging effect.
  ● Melatonin: The indolic hormone melatonin, which is widely present in various tissues including the skin, regulates circadian rhythms and promotes sleep. Melatonin can penetrate membranes and mitigate lipid peroxidation and protein oxidation, as well as oxidative damage to the mitochondria and DNA caused by UVR [59]. In a randomized, split-face, assessor-blinded, prospective 3 month study, 22 women (mean age 55 years) with moderate to severe skin aging applied melatonin-based creams to their faces. The melatonin-based creams significantly improved the skin tonicity and skin hydration with a significant reduction in the skin roughness [60]. This suggests the skin anti-aging effect of melatonin.
  ● Protocatechuic acid: Protocatechuic acid has antioxidant and anti-inflammatory effects. This is caused by Akt, mTOR, and NF-κB in HaCaT cells and the primary keratinocytes pathway plays a role, which is also regulated by JNK and p38 MAPK [61]. Female subjects (n = 22) applied 0.02% protocatechuic acid for 8 weeks to address facial wrinkles, crow’s feet around the eyes, and other MMP-1 secretions. The results showed that protocatechuic acid could inhibit MMP-1 produced by irradiation and inhibit the percentage of skin wrinkles [62]. This revealed that protocatechuic acid has the effect of skin anti-aging.
  ● Nature-Based bakuchiol: Natural bakuchiol has a strong antioxidant activity, which can significantly reduce the levels of PGE2 and MIF [63]. Female subjects (n = 60) with sensitive skin (e.g., eczema, blackheads, and cosmetic intolerance) were selected and they applied cosmetics containing natural bakuchiol according to the needs of the face for 4 weeks. The smoothness, gloss and overall appearance of a subject’s skin after 4 weeks were detected, and the results showed that natural bakuchiol had a therapeutic effect on patients with sensitive skin [64]. This suggests that natural bakuchiol has certain anti-aging effects on the skin.
  ● Rhamnose: Rhamnus can inhibit the generation of AGEs and the expression of MMP-9 [65,66]. Healthy women (n = 20) aged 50–68 were selected as participants, and a 5% rhamnose emulsion with a 5% was applied, twice a day, to the forearms of each participant for 8 consecutive weeks. The results showed that rhamnose can promote an increase in the skin epidermal thickness and collagen production [67]. This indicates that rhamnose has the potential to improve skin aging.
  ● Timosaponin AIII (TA-III): Timosaponin A-III are steroidal saponins extracted from the traditional Chinese medicine, Anemarrhena asphodeloides, which has anti-inflammatory activity [68]. Moreover, the transcriptional levels and protein expressions of cyclooxygenase-2 (COX-2) and MMP-9 induced by UVB were inhibited in a dose-dependent manner. The TA-III inhibited UVB-induced protein kinase (MAPK) signaling, protein-1 (AP-1), and nuclear factor κB (NF-κB) activation, thereby preventing a TNF-α overexpression of interleukin-6 (IL-6) and COX-2 in human epidermal keratinocytes [69]. Female subjects (n = 21) aged between 43 and 55 years old, who had begun to form or had already-formed wrinkles and who met the selection criteria of subjects were selected, and the agent containing 0.25% of TA-III was applied for 12 weeks. The results showed that wrinkles were significantly reduced, and that secreted MMP-1 was reduced, which had the effect of resisting photoaging [70]. This revealed that the TA-III had the effect of treating skin aging.
  ● Poly L lactic acid (PLLA): PLLA not only supports an increase in HIF-1α but also promotes collagen synthesis [71]. Healthy women (n = 40) were selected randomly and received treatment three times every 4 weeks. They were injected with PLLA on both sides of the face (prepared in a 1:1 dilution ratio of sterile water and PLLA), with 5 to 6 mL on each side. A test was conducted after completion of the experiment. The results showed that the PLLA treatment group showed an increase in skin elasticity and moisture content, a decrease in skin epidermal moisture, and a significant reduction in pigmentation, erythema, and pore size [72]. This indicates that PLLA has an anti-aging effect on the skin.
  ● 10-Hydroxystearic Acid (10-HAS): 10-HSA is the most effective PPAR α Agonist, and it increases the level of type I collagen in primary human fibroblasts [73]. Forty-two Caucasian women with Fitzpatrick skin types II-III were treated with 1% (33 mM) HSA twice daily for 8 weeks. The results showed that 10-HSA can reduce the expression of the aging protein p53, inhibit the expression of the MMP-1 gene, reduce the pigmentation of senile plaques, and improve the coarseness of pores [74]. This reveals the anti-aging effect of 10-HSA on the skin.
  ● Glycolic acid (GA): GA can reduce UVB-induced cellular inflammation and aging, regulate the expression of MMPs and collagen, and reduce the expression level of MMP-9 induced by UV. GA can be more widely used for photoaging and inhibit the formation of wrinkles [75]. A total of 262 randomly-selected healthy women aged 30 to 60, with moderate to late stage photoaging skin, and a 70% glycolic acid facial peel was applied on their faces. After 14 days of the experiment, it was found that the subjects who applied glycolic acid had a better skin tolerance and resulting cosmetic effects compared to those who did not apply glycolic acid [76]. This reveals that organic acids such as glycolic acid may have anti-aging and skin condition-improving effects on the skin.

The compounds with anti-aging effects on the skin studied in clinical trials on human subjects are briefly presented in Table 1

◆ Data from Animal experiments:
  ● Bavachalcone: Bavachalcone is a prenylchalcone that is the major bioactive chalcone isolated from *Psoralea corylifolia* [77,78]. The experiment was conducted using WT and TET3+/− C57BL/6J mice, where paraquat (at 10 mg/kg, with a dosage volume of 0.1 mL/10 g mouse body weight) was intraperitoneally injected for modeling and administration. The paraquat treatment was carried out from the sixth week until the fortieth week, while simultaneously, bavachalcone (at 20 mg/kg, with a dosage volume of 0.05 mL/10 g mouse body weight) was administered by gavage. The results showed that, in comparison with the control group, the activity of SOD in the skin tissue of mice in the treatment group was lower; the MDA levels decreased; the activity of catalase (CAT) increased; and the content of Hyp increased [77,79]. This reveals that bavachalcone has an anti-aging effect on the skin.
  ● Banana peel polyphenols (BPP): Banana peel polyphenols have antioxidant effects [80], and an experiment was carried out with rats where the model was made by a subcutaneous injection of 300 mg/kg d-galactose for 7 weeks. An amount of 120 mg/kg BPPs was administered by gavage for 7 weeks. An analysis of the experimental results showed a significant increase in the content of type I and II collagen in the skin tissue, a decrease in the expression level and protein content of MMP-1, and an increase in the expression level and protein content of the AQP gene [81]. This indicates that BPPs have a therapeutic effect on skin aging.
  ● Curcumin: Curcumin is a component of curcuma [82]. Curcumin can block the signal pathway of light damage, and it has the effect of resisting oxidative stress and inhibiting inflammation [83]. BALB/c-nu/nu mice were used to construct a skin aging model, and 125 mg/kg/d d-galactose was injected subcutaneously to create this skin aging model for 21 consecutive days. After modeling, 80 mg/kg of curcumin was administered by gavage for three consecutive weeks. After the experiment, the results for a skin tissue detection showed an increase in the SOD activity and Hyp content accompanied by a decrease in the MDA content [84,85]. This study demonstrates that curcumin can significantly affect skin aging-related indicators. Moreover, it has the ability to resist skin aging.
  ● Procyanidins: Procyanidins have therapeutic potential for photoaging, with antioxidant and anti-inflammatory effects, and they can inhibit the expression of MMPs induced by UVB [86]. Kunming female mice were injected with 5% d-galactose 10 mL/kg subcutaneously every day, UV radiation exposure was conducted the next day, and then 50 and 100 mg/kg of procyanidins were administered by gavage for 30 consecutive days. An analysis of the skin tissue indicators showed a decrease in the MDA levels; and an increase in the SOD value and GSH-Px activity and inhibiting oxidative damage [87,88,89]. The results imply that procyanidins have therapeutic effects on skin aging.
  ● Resveratrol: Resveratrol is a polyphenolic compound that has therapeutic effects on skin photoaging. It exerts anti-aging effects on the skin through various pathways such as MAPK, MAPKK, FOXO3, TGF, or MMP-1 [90]. Using adult Wistar female rats, bilateral ovaries were excised to reduce the secretion of estrogen, and a skin aging model was constructed. Resveratrol was administered orally at doses of 10, 30, and 60 mg/kg for 31 consecutive days to detect skin tissue indicators. The results showed an increase in the SOD and hydroxyproline (Hyp) contents, a decrease in the malondialdehyde (MDA) content, and an increase in the SIRT1 protein content [91], indicating that resveratrol has an anti-aging effect on the skin. In addition, studies have shown that resveratrol also has an anti-scar formation effect [92].
  ● Codonopsis pilosula polysaccharides (CPP): Codonopsis pilosula polysaccharides are one of the main active ingredients of Codonopsis pilosula. Codonopsis pilosula polysaccharides have antioxidant, anti-inflammatory and anti-aging effects [93]. This experiment was conducted on mice. The model was made by a subcutaneous injection of a 5% d-galactose solution 1 g/kg for 42 consecutive days, and Codonopsis pilosula polysaccharide of 50 mg/kg and 150 mg/kg was administered by gavage for 32 consecutive days. An analysis of the skin tissue indicators showed a decrease in the MDA levels [93,94]. This implies that CPPs have anti-aging effects on the skin.
  ● Lycium barbarum polysaccharide (LBP): LBP is a natural polysaccharide extracted from Lycium barbarum, which has anti-inflammatory, anti-apoptotic, and anti-aging effects. Its main effects include reducing ROS and MDA while increasing SOD in the body [95]. These effects are essential for the resistance of photoaging [96]. This experiment was conducted with mice, and the skin aging model was established by a subcutaneous injection of 1000 mg/kg/d d-galactose for 42 consecutive days. Low, medium, and high doses of 5, 10, and 20 mg·kg-1·d-1 were administered by gavage. A daily dosage of 20 mL per kilogram of mouse weight was administered for 42 consecutive weeks. After 42 days, the SOD activity and GSH-Px activity in the skin tissue of the mice were significantly increased. Studies have shown that MDA is an important indicator for detecting skin aging, exhibiting a decrease in the MDA content and an increase in the Hyp content [97,98]. This indicates that LBP can resist skin aging.
  ● Saussurea Medusa Maxim Polysaccharides (SMMP): Saussurea Medusa Maxim Polysaccharides have anti photoaging effects [99]. This experiment was conducted on mice. The model was created by subcutaneously injecting d-galactose and subjecting the mice to 5% UVB radiation, with a daily dose of 10 mL/kg of D-galactose. The model was established for 40 consecutive days, and starting from the 11th day of the experiment until the end, the mice were administered 4 g/kg/d SMMPs by gavage. After the completion of the modeling, the indicators of skin tissue were tested, and the results showed a significant increase in the SOD activity, GSH-Px activity, and Hyp activity, but they showed a decrease in the MDA content [100]. This indicates that SMMPs have anti-skin aging effects.
  ● Pyrroloquinoline Quinone (PQQ): PQQ has an inhibitory effect on the secretion of MMPs and can prevent a decrease in collagen synthesis [101]. Wild-type mice (WT) and Bmi-1 gene knockout homozygotes were utilized to create a mouse model. The Bmi-1 gene, associated with aging, was knocked out to induce the aging phenotype. An amount of 4 mg of PQQ was added to each kilogram of feed and raised for 4 weeks. The experimental results showed an increase in collagen, and enhanced skin cell proliferation, reduced skin tissue fibrosis, cleared oxygen free radicals, and decreased ROS levels [102]. This indicates that PQQ has an anti-aging effect on the skin.
  ● l-linalool: l-linalool has sedative and anxiolytic effects [103]. Female mice of a healthy Swiss species (ICR strain) were injected subcutaneously with 1.5 mg/g/d d-galactose for the construction of a skin aging model for 30 consecutive days. An amount of 5.0% of l-linalool was administered to the skin tissue. The skin tissue indicators were detected, and the results showed that the SOD increased, MDA decreased, skin moisture increased, Hyp content increased, oxygen free radicals were eliminated, and lipid peroxidation was reduced [104]. This indicates that the drug has anti-aging effects on the skin.
  ● β-Nicotinamide Mononucleotide (NMN): NMN is currently a component that can treat oxidative stress-induced skin aging [105]. This experiment involved the construction of a mouse model of photoaging, exposing the mice to UVB radiation. The skin’s oxidative damage was induced using UVB (400 nm), and the mice were subjected to 12 h of UVB irradiation per day for 1 week. The use of 300 mg/kg bw of NMN was used for intraperitoneal injection for three consecutive weeks. The experimental results indicated that NMN can reduce inflammatory factors (e.g., TNF-α, IL-1 β, IL-6, etc.) and improve SOD levels. This indicates that NMN has a therapeutic effect on skin aging [106].
  ● Quercetin: Quercetin is a natural polyphenol with antioxidant and anti-inflammatory effects, which can inhibit the expression of MMP-1. Quercetin significantly inhibits the activity of AP-1 and NF-κB nuclear factors induced by ultraviolet radiation. It can directly target PKCδ and JAK2 in the skin, and it has a protective effect on UV-mediated skin aging and inflammation [107]. Male Kunming mice were injected with d-galactose at a concentration of 200 mg/kg/day for 8 weeks, and 10–20 mg/kg/day quercetin was injected subcutaneously for 8 weeks. By detecting a degree of looseness and roughness of the skin, a thickening of the epidermis and dermis, an increased collagen content, a decreased expression of aging proteins, and an increased superoxide dismutase (SOD), total antioxidant capacity (T-AOC), and glutathione peroxidase (GSH-Px) [108], the study demonstrated that quercetin has an anti-aging effect on the skin.
  ● Vitamin E: Vitamin E is an efficient antioxidant that can resist skin photoaging, inhibit the production of oxygen free radicals, and protect the skin from the adverse effects of photoaging. Vitamin E is a positive drug that inhibits skin oxidative aging. This experiment involved homogenizing Hainan pig skin and subsequently adding 20–100 μL of vitamin E (≥98%) for 1 h of a dark reaction. The results indicated a significant increase in both the SOD activity of the skin tissue and CAT activity; it also showed a reduction in the content of protein oxidation products caused by hydrogen peroxide [109]. This demonstrates that vitamin E has an anti-aging effect on the skin.
  ● Chitosan: Chitosan can resist oxidation and inflammation, maintaining the form and level of collagen [110]. Experiments were conducted using rats where chitosan solution with a mass fraction of 1% was applied daily. The results showed an increase in the Hyp content in the skin tissue and a denser collagen fiber [111,112]. Hyp is a unique amino acid for synthesizing collagen, indicating that chitosan can promote skin firmness and have anti-aging effects on the skin.
  ● Ganoderma lucidum polysaccharides (GLP): Ganoderma lucidum polysaccharides are natural antioxidants with no toxic side effects, which can counteract the B-induced photoaging of fibroblasts. Ganoderma lucidum polysaccharides can inhibit the production of ROS [113]. This experiment was conducted on mice, and the model was made by a subcutaneous injection of 1000 mg/kg/d d-galactose for 42 consecutive days. A daily dosage of 0.5% ganoderma lucidum polysaccharide was applied to skin tissue for 42 consecutive days. After the completion of the modeling the relevant indicators of skin tissue were tested, and the results showed that ganoderma lucidum polysaccharides can eliminate oxygen free radicals, reduce the MDA content, improve the SOD activity and increase the Hyp content of collagen [114]. This reveals that Ganoderma lucidum polysaccharides can resist skin aging.
  ● Wax gourd polysaccharides (WGP): Wax gourd polysaccharides have antioxidant effects and can scavenge oxygen free radicals [115]. This experiment was carried out on mice. The model was made by administering 500 mg/kg/d d-galactose for 6 weeks, and then the skin tissue was smeared with 5 mg/mL of wax gourd polysaccharide every day, using 2 mL every day, for 6 weeks. After 6 weeks of administration, the WGPs had the ability to scavenge hydroxyl radical and superoxide anion radicals; moreover, at 2 mL, it had an inhibitory effect on tyrosinase. After testing the skin tissue-related indicators, the SOD activity and CAT activities increased while the MDA content decreased [116]. This indicates that WGPs have anti-aging effects on the skin.
  ● Hydroxyacetic acid: Hydroxyacetic acid has the effect of slowing down skin aging, reducing cellular inflammation caused by UVB, resisting photoaging, and inhibiting the expression of MMP-9 [50]. Kunming mice were injected subcutaneously with 1 mg/g/d d-galactose for 12 weeks. Hydroxyacetic acid with concentrations of 20%, 35%, 50%, and 70% was applied every 2 weeks for administration. The relevant indicators of the skin tissue were tested, and the results showed an increase in collagen and elastic fibers [75,117]. This indicates that HA has a certain effect on resisting skin aging.

The review of skin anti-aging compounds studied in animal experiments is briefly presented in Table 2.

◆ Data from experimental studies on cell cultures:
  ● GX-50: GX-50 is an extract from Zanthoxylum bungeanum [119]. Primary human skin fibroblasts were utilized for this modeling, with a treatment involving 50 μM of hydrogen peroxide. The treatment was administered once every two days, totaling four times. GX-50 was also administered once every two days for a total of four times. After the experiment, an analysis of a β-Galactosidase staining test showed that the positive rate was reduced and the MDA content or ROS level was decreased [120]. This indicates that GX-50 has a certain therapeutic effect on skin aging.
  ● Ligustrazine: Ligustrazine has good antioxidant activity and has the potential to treat skin aging caused by ultraviolet radiation [121]. Using UVA irradiation, human skin fibroblasts were used to create a skin aging model, using 10 J per day for five consecutive days, where a ligustrazine hydrochloride injection (2 mL:40 mg) was administered for the five consecutive days. The pharmacological effect of the ligustrazine on skin aging was tested, and the results showed that the positive rate of β-galactosidase staining was decreased, the total amount of ROS was decreased, the expression level of the p53 protein was decreased, and the mRNA expression levels of MMP-1 and MMP-3 were decreased [122]. The experimental results indicate that ligustrazine has an anti-aging effect on the skin.
  ● Caffeic acid phenethyl ester (CAPE): CAPE is the active ingredient of propolis [123]. Human dermal fibroblasts (HDF) were selected and used to construct an aging model using ultraviolet radiation. The results of administering CAPE showed that CAPE can inhibit the expression of MMP-1 in human skin cells, and the pathway of action occurred when the CAPE inhibited lysine acetylation induced by UV in human skin tissue [124]. This reveals that CAPE has the effect of resisting skin aging.
  ● Total flavonoids of Eurasian Convolvulus: A skin aging model was induced using mouse skin fibroblasts and 0.1–0.2 g/L of AGEs. The induction process lasted for 48 h, and the skin aging model was induced. Afterwards, 0.1–0.4 g/L of total ketone of Eurasian cyclodextrin was administered for a continuous duration of 6 h. Then, pharmacodynamic testing was conducted, and the experimental results showed that the total ketones of Eurasian spiny flowers can inhibit the specific receptors of glycation end products [125]. This indicates that the total flavonoids of Eurasian Convolvulus can prevent skin aging.
  ● Epimedin C: Epimedin C is the major flavonoid from *Herba epimedii* [126]. HaCat cells were employed to construct a skin aging model in which AGEs were used. The HaCat cells were treated with a concentration of 1000 μg/mL of AGEs to create the skin aging model. Additionally, the drug was administered to the HaCat cells at a concentration ranging from 100 to 300 μM. Pharmacodynamic testing was performed after 24 h of administration. The results showed that Epimedin C can reduce ROS, inhibit the abnormal expression of the MMPs family, inhibit an increase in the apoptotic protein Bax, inhibit an increase in cyclooxygenase Cox-2, and inhibit the expression of IL-6 and IL-8 [127]. This suggests that Epimedin C has an anti-aging effect on the skin.
  ● Schisandrin A, Schisandrin B, and Schisandrin C: Schisandrin B has a protective effect against skin tissue oxidative stress induced by solar radiation and can prevent skin photoaging [128]. Using HaCat cells for experiments through 50–200 μM of hydrogen peroxide for modeling, a model was established after 4 h. Concentrations of 100–200 μM of Schisandrin A, Schisandrin B, and Schisandrin C were administered for 12 h. Oxidative damage reactions were tested to determine the cell survival rate and the experimental results showed that Schisandrin A, Schisandrin B, and Schisandrin C had a certain protective effect against oxidative damage [129]. This indicates that Schisandrin A, Schisandrin B, and Schisandrin C have certain anti-aging effects.
  ● Cordycepin (3-deoxyadenosine): Cordycepin has anti-inflammatory effects. It was used to create a UVB model of human dermal fibroblasts, lasting for 24 h, which were then administered with cordycepin. The experimental results showed that 50 μM and 100 μM of cordycepin can reduce the expression of MMPs and inhibit the activation of NF-κB to prevent skin photodamage caused by UVB [130]. This indicates that cordycepin has an anti-aging effect on the skin.
  ● Astragaloside IV: Astragaloside IV is one of the main active ingredients extracted from Astragalus membranaceus. It has the effect of resisting light aging [131]. Human skin fibroblasts were utilized, specifically by selecting aged skin fibroblasts derived from elderly individuals. These aged skin fibroblasts were then treated with a concentration of 5–20 μg/mL of astragaloside IV and monitored over a duration of 24–120 h. The experimental results showed that the astragaloside IV promoted collagen synthesis in skin cells and reduced the cell apoptosis rate [132]. This indicates that astragaloside IV has a certain anti-aging effect.
  ● Ginsenoside Rb1: Ginsenoside Rb1 has various pharmacological effects, including anti-inflammatory, anti-stress, anti-aging effects, etc. [133]. This experiment was conducted using human dermal fibroblasts and hydrogen peroxide (600 μM) for modeling, with a modeling time of 3 h. After 3 h of modeling, Ginsenoside Rb1 (500 μM) was added for 2 h. The results demonstrated that the Ginsenoside Rb1 exhibited a significant resistance to the toxicity of hydrogen peroxide, and embodied a mechanism of action to reduce the ROS value [134,135]. This implies that ginsenoside Rb1 can resist skin aging.
  ● Vitamin D3: Vitamin D3 is produced in the skin from 7-dehydrocholesterol [136]. Vitamin D3 and its active metabolites can effectively protect against photodamage, reducing skin aging through multiple mechanisms, including immune regulation and DNA repair [137]. Human epidermal keratinocytes were used for modeling through UVB (50 mJ/cm^2^), and the cells were pre-treated with active vitamin D3 and L3 hydroxyderivatives for 24 h. By activating Nrf2, which functions in cytoprotection and detoxification, these compounds reversed the UVB-mediated ROS production, thus attenuating photoaging. These compounds also stimulated the expression of antioxidant-response genes, including GR, HO-1, CAT, SOD-1, and SOD-2, as well as the expression of HO-1, CAT, and MnSOD proteins [138]. This indicates that vitamin D3 promises to be a skin photoprotector.
  ● Metformin: Metformin can repair DNA damage, clear ROS, and protect cells [139]. Human skin fibroblasts were used for modeling through UVA, and metformin with a drug concentration of 1 mmol/L was used for 72 h of a drug treatment. An analysis of the skin aging-related indicators showed that the positive rate of β-galactosidase staining decreased, the ROS levels decreased, the expression levels of MMP-1 and MMP-3 decreased and the expression levels of SOD1 and SOD2 also increased [140]. This indicates that the degree of skin aging had improved.

A summary of skin anti-aging compounds studied in cell culture experiments is briefly shown in Table 3.

## 4. Discussion

At present, the research of skin aging and the development of anti-aging products and related compounds are developing rapidly. We have reviewed the recent progress of the above active compounds and aging model selections on skin anti-aging and, accordingly, the tactics taken, which are discussed as follows.

### 4.1. Clinical Studies on Human Subjects

In the clinical studies on human subjects, studies with female participants were more common, with 88% of all the experiments selecting only female subjects. The number of the selected subjects ranged from 20 to 262, taking into account the randomness and individual differences of the experiments. The selected subjects’ ages ranged from 18 to 68 years old. Among the subjects, 38% of them were chosen for exhibiting signs of aging skin. The advantage of directly selecting these subjects is that it reduces the time required to create a skin aging model; however, one disadvantage is that the mechanism of skin aging is not clear, making it difficult to explore the drug’s target based on a specific mechanism. Another limitation is that many studies only involve patients from specific geographic regions, genders, and age groups.

When choosing the method of administration, whether to use a topical application, injection, or oral administration is an important consideration in clinical studies on human subjects. The chemical properties of a drug should be taken into account when selecting the method of administration. For example, orally-administered hydrolyzed collagen protein is absorbed in the small intestine, producing high concentrations of Gly-Pro-Hyp and its hydrolyzed forms, which enters the bloodstream and can be transferred to the skin to exert their effects [142]. In contrast, drugs applied topically to the skin are mainly absorbed through the stratum corneum, hair follicles, sweat glands, and sebaceous glands, without entering the systemic circulation [143]. The choice between a topical and oral administration of a drug depends on its ability to penetrate the outer layer of the skin, which is influenced by various factors such as the physical and chemical properties of the substance (e.g., molecular size, stability, binding affinity, and solubility), the thickness and composition of the skin, the skin metabolism, the location, the region, and the duration of an application.

In these clinical studies, the types of active ingredients used were mostly natural compounds, and the majority of administration methods involved a topical application. The duration of the topical administration was mostly 8 ± 4 weeks, and the tactics involved mainly included antioxidant, anti-inflammatory, and collagen supplementation. We admit that we only conducted a mini review on skin anti-aging products and some clinical data may not be involved. Future research needs to focus on addressing the safety and efficacy of these compounds in humans through more clinical trials.

### 4.2. Animal Experiments

In animal experiments, rats and mice are the most commonly used animals. Among these studies, 76% of researchers mainly used mice, while only 17% used rats, and all three of those studies used SD rats. The most widely used mouse strain was Kunming mice (41%). A total of 11% of the studies used male animals, 64% of studies used female animals, and 11% of studies used both male and female animals, while 11% of the studies did not report the gender.

Animal experiments can avoid clinical, ethical problems and provide important references for clinical studies. Different animal aging models have their own advantages and disadvantages and are applied in different research fields. It is recommended to choose an animal aging model that best fits the experimental needs based on the research objectives and design.

When creating animal aging models, the use of d-galactose to induce aging models has been widely used, with injection being the main method of administration. The advantages of this method are that this model can well reflect the aging nature induced by increased oxidative stress, the modeling time is relatively short, the repeatability is good, and the survival rate is high. The disadvantage is that there are differences in the immune and biochemical indicators between induced aging and natural aging.

When selecting the dosage for creating aging models, the dosage range of d-galactose was 125 to 1500 mg/kg, and the subcutaneous injection of 1000 mg/kg of d-galactose was the most commonly used dosage in the experiments. The administration time ranged from 3 to 12 weeks. Studies have shown that in rats, a subcutaneous injection of d-galactose (at 125–300 mg/kg per day) usually requires 6 to 10 weeks to establish a stable aging model, while the intraperitoneal injection of d-galactose (at 100–300 mg/kg per day) requires 4 to 7 weeks [144]; however, most of the dosages used in the studies reviewed in this article differed significantly from the recommended dosages, indicating that although this model has been widely used, there is no unified standard for model establishment and evaluation. The dosage and duration of modeling used in different laboratories, therefore, can vary significantly, considering factors such as the type and weight of the experimental animals, the method and duration of administration, the experimental conditions, and the potential adverse reactions when choosing a dosage.

Animal aging models can also be created using methods such as gene knockout. The disadvantage of this is that it differs from natural aging and costs more. This method can be used to investigate the effects of individual genes on aging. For example, in mice with a Bmi-1 gene knockout, the mitochondrial function is severely damaged, leading to a significant increase in ROS levels. Using Bmi-1-deficient mice can be used to study the effects of anti-aging drugs in inhibiting oxidative stress; however, an ideal animal model can still be further improved.

The types of drugs used in animal aging models were mostly natural compounds, and the most common method of administration was oral administration. The pathways involved mainly included antioxidant, anti-inflammatory, and collagen supplementation. Many natural compounds with potential anti-aging effects have been discovered, such as polyphenols (i.e., resveratrol, anthocyanins, curcumin, etc.) and polysaccharides (CPP, LBP, SMMP, etc.), as well as synthetic small-molecule compounds such as hydroxy acids, which have shown significant anti-aging effects in animal models.

### 4.3. Experimental Studies on Cell Cultures

In the experimental studies on cell cultures, 90% of the cells used were human skin cells, and 10% were mouse skin cells. Among the human skin cells used in the experiments, 70% were human fibroblasts. There were also studies using immortalized human epidermal cells and human epidermal keratinocytes. All of the mouse cells used were fibroblasts.

In creating the cell aging models, the commonly used modeling agents included UV irradiation, hydrogen peroxide, and advanced glycation end products (AGEs). A total of 27% of the models were created using hydrogen peroxide, with a concentration of 50–600 μM. A total of 50% of the models were created using UV radiation, with a required UV dose of 5–10 J/d, while 23% of the models were created using AGEs, with a dosage of 0.1–0.2 g/L.

The most commonly used method for constructing an in vitro model of skin aging is to use UV radiation, mainly using HSF cells and HaCaT cells. Human skin fibroblasts (HSF) are mainly located in the dermis (with a small number present in the epidermis), while human skin keratinocytes (HaCaT) are located in the epidermis. Since UVB primarily damages the skin’s epidermis, while UVA can reach the dermis, it is common to use UVA and UVB irradiation separately on HSF cells and HaCaT cells to construct a model [145]. After irradiation with different doses, the relevant aging markers are observed to determine if a model has been successfully constructed. Since UV irradiation can damage cells, the distance between the light source and the cells, as well as the irradiation time, need to be considered to achieve the appropriate level of cell damage. The literature reviewed in this article suggested that the UV dose was generally chosen in the range of 5–10 J/d. In actual experiments, a series of gradient UV doses are usually set and irradiated separately to obtain dose groups with *p* < 0.05 or *p* < 0.01, and the selected dose is then used for the subsequent induction of aging.

Although the cell aging model can control the experimental factors well, and it is also rapid, convenient and has a high through-put, it has certain limitations because it is an in vitro environment and may differ from the situation of aging in vivo. In addition, most of the drugs used to test skin aging effects on cells come from natural compounds, with only 10% coming from synthetic compounds. These tested compounds still raise some concerns about their bioavailability in the body or permeability in the skin and need further identification in the future.

### 4.4. Skin Anti-Aging Tactics

Skin aging is a complicated pathophysiological process and is caused by both intrinsic and extrinsic factors. Intrinsic aging is an inevitable physiological process, while extrinsic aging is caused by external environmental factors such as air pollution, smoking, malnutrition, and sunlight exposure, leading to a loss of elasticity and rough texture.

One of the main theories of skin aging is that skin aging is usually caused and affected by increased oxidative stress [146]. The best-used anti-aging tactics in skin is to use antioxidants. Taking appropriate antioxidant medications can help eliminate the effects of oxidative stress. On one hand, most of the anti-aging products used in skin can reduce the ROS value, inhibit the MDA level, reduce the lipid peroxidation ability, and reduce the protein oxidation. These activities may be associated with an increased ability to scavenge oxygen free radicals. On the other hand, many compounds or drugs can promote the activities of SOD, CAT, and GSH-Px and it is indicated that these components or drugs can enhance the antioxidant capacity.

The antioxidant ingredients mainly include sterols (e.g., triterpenoid saponin), chalcones, diketones, polyunsaturated hydrocarbons, flavonoids, linalools, non-flavonoid polyphenol organic compounds, acids, guanidine, quinoline quinones, catechins, polyphenols, natural polysaccharides, pyrazine, phenylalkyl glycosides, enols, and lignans. The main functional chemical structures are a degree of un-saturation and active chemical bonds containing hydroxyl and other chemical groups, or a ketone structure and polysaccharide structure, which play an important role in scavenging oxygen free radicals and enhancing the antioxidant defense. Most of these compounds come from nature and have less safety issues.

Another important factor contributing to skin aging is sunlight and UV exposure. Light damage to the skin is the main factor leading to exogenous skin aging [147]. Excessive exposure of the skin to UV radiation can trigger an immune system remodeling and can lead to photoaging; therefore, it is important to avoid excessive exposure to sunlight or UV lamps [148]. Many products focused on UV protection against skin aging have been well developed. Additionally, besides the natural products, some compounds, such as vitamin D and melatonin are produced by the skin and also exert a variety of antiaging and photoprotective effects on the skin. Vitamin D and its active metabolites exert photoprotective effects to reduce premature skin aging and cancer through various mechanisms such as immune regulation and DNA repair. They interact with a variety of nuclear receptors, including the vitamin D receptor, aryl hydrocarbon receptor, liver X receptors, and the retinoic acid-related orphan receptors α and γ, through which skin aging can be prevented, mitigated, or treated [137]. Melatonin plays a photoprotective role against skin aging by promoting DNA repair and reducing the inflammatory response [59].

In addition, some compounds or drugs can exert anti-wrinkle and anti-aging activities by promoting collagen synthesis and inhibiting collagen degradation, while some compounds or drugs can directly increase the Hyp level and collagen content in the skin. Moreover, some compounds or drugs have significantly decreased the levels of MMPs in skin and it is indicated that these products can decrease the degradation of collagen in the skin.

Both inflammation and glycation are very important in the development of skin aging. Some compounds have showed related anti-aging activities working via anti-inflammatory or anti-glycation mechanisms in the skin; however, the number of these compounds are still relatively small and need to be further validated and updated in the future.

In addition to the several mainstream theories mentioned in this article, other skin aging theories are also emerging and keep on updating our knowledge of skin aging. Molecular and cellular biology both play an important role in exploring the mechanisms of skin aging and in looking for new targets. Recently, emerging studies have indicated that the skin’s functions are regulated by the cutaneous neuroendocrine systems. The skin can produce hormones, neuropeptides and neurotransmitters including vitamin D3 and melatonin which may induce/stimulate downstream signaling through the activation of corresponding receptors and by exerting a regulatory effect on skin aging [149]. The mitochondrial aging theory has also become an increasingly accepted theory [150]. There are also proposals for hypoxia induction and targeted anti-aging repair systems in the field of anti-aging in skin [151]; however, suitable compounds or drugs are still lacking.

In the field of cosmetics, based on the skin aging theory, there are specific anti-aging tactics in skin being taken. Antioxidant, UV-protection, anti-collagenase, anti-elastase, anti-hyaluronidase, anti-tyrosinase and anti-senescence effects including the inhibition of matrix metalloproteinase production, and anti-inflammatory, anti-glycation and moisturizing activities in skin cells, could be used to determine the direct or indirect effects of aging in the skin [37,152,153].

In the coming studies, according to the skin aging mechanisms and targets, a chemical separation and synthesis, biological and chemical engineering, and synthesis biology may provide plentiful sources for identifying new active ingredients. In the field of cosmetics and pharmaceutics, new drug delivery systems or formulas are expected to improve the pharmacokinetic behaviors of active ingredients in the skin. In the present review, few products used a new drug delivery system to improve the pharmacokinetic behaviors. In the future, with the occurrence of new skin aging theories, and anti-aging ingredients, as well as treatment methods and preparations, anti-aging products in the skin will become increasingly abundant or revolutionary.

## 5. Conclusions

Skin aging has become an increasingly important topic of concern. This review summarized the effective drugs for skin aging in human, animal, and experimental studies on cell cultures. There has been great progress in skin aging, but ideal drugs are still lacking or need further validation. One of the key mechanisms of skin anti-aging compounds is to eliminate ROS and reduce skin oxidation, and the current compounds are mostly natural products, with fewer biochemical products. The strategies and productions of anti-aging in the skin still need to be further improved in the future by interdisciplinary technologies. In the future, we should further investigate new mechanisms for skin aging, we should identify new active ingredients including biochemical products, and we should develop safer and more effective administration methods with a higher bioavailability or permeability for relevant experimental objects.

## Figures and Tables

**Table 1:** Summary of skin anti-aging drugs/ingredients studied on human subjects.

No.	CAS No.	Compound Category	Classification of Compound Structures	Drug/ Compound	Subjects	Administration	Mechanisms	References
Oral administration (dietary)								
1	24696-26-2	Natural compounds	Amides	Ceramide	30 adults aged 20–60 (men and women)	4 w	Skin barrier↑	Tsuchiya, 2020 [42]; Tsuchiya, 2021 [43]
2	68-26-8	Natural compounds	Enols	Retinol	24 women aged 18–65 with acne, etc.	6 w	Collagen↑	Shao, 2017 [44]; Sadick, 2019 [45]
3	9004-61-9	Natural compounds	Saccharide	Hyaluronic acid	60 women with aging skin	12 w	Collagen integrity↑	Yoshida, 2019 [46]; Michelotti, 2021 [47]
4	472-61-7	Natural compounds	Terpenes	Astaxanthin	23 healthy women	10 w	Oxidation↓, inflammation↓	Zhou, 2021 [48]; Iwona, 2021 [49]; Ito, 2018 [50]
5	2239-67-0	Synthetic compounds	Peptides	Gly-Pro-Hyp	64 women	12 w	Collagen degradation↓	Chae, 2023 [51]; Kim, 2018 [52]
Topical administration or injection								
6	14024-63-6	Natural compounds	Aminoketones	AZ	31 healthy women aged 44 ± 7	8 w, applied to face	ROS↓	Swindell, 2020 [53]; Dhaliwal, 2021 [54]
7	989-51-5	Natural compounds	Catechols	EGCG	88 women	12 w, applied	ROS↓, DNA loss↓	Tong, 2014 [56]; Lei, 2023 [55]
8	78619-96-2	Natural compounds	Esters	Vitamin C	50 women	4–8 w, applied to face	ROS↓	Akbari, 2014 [57]; Rattanawiwatpong, 2020 [58]
9	73-31-4	Natural compounds	Indoles	Melatonin	22 women with moderate-severe skin aging	12 w, apply to face	ROS↓	Bocheva, 2022 [59]; Milani, 2018 [60]
10	99-50-3	Natural compounds	Phenolic acids	Protocatechuic acid	22 women	8 w, applied to face	Oxidation↓, inflammation↓	Shin, 2020 [62]; Nam, 2018 [61];
11	10309-37-2	Natural compounds	Phenols	Nature-based bakuchiol	60 women with sensitive skin	4 w, apply to face	Oxidation↓	Bluemke, 2022 [63]; Draelos, 2020 [64]
12	6155-35-7	Natural compounds	Saccharide	Rhamnose	20 healthy women aged 50–68	8 w, applied to forearm	AGEs↓	Péterszegi, 2008 [66]; Robert, 2010 [65]; Pageon, 2019 [67];
13	41059-79-4	Natural compounds	Saponins	TA-III	21 women who had started to develop or had already developed wrinkles	12 w, applied	Inflammation↓, photoaging↓	Im, 2020 [70]; Lin, 2020 [68]; Kim, 2019 [69]
14	26811-96-1	Synthetic compounds	Esters	PLLA	40 Healthy women	12 months, injected in the face	Collagen synthesis↑	Oh, 2023 [71]; Bohnert, 2019 [72]
15	638-26-6	Synthetic compounds	Fatty acids	10-HSA	42 Caucasian women with Fitzpatrick skin types II-III	8 w, applied to face	Collagen↑	Rawlings, 2021 [73]; Schütz, 2019 [74]
16	79-14-1	Synthetic compounds	Hydroxyl acids	GA	262 women aged 30–60 with photoaging skin	2 w, applied to face	Inflammation↓, photoaging↓	Tang, 2019 [75]; Santos-Caetano, 2020 [76]

↑ or ↓ indicates increased or decreased, respectively.

**Table 2:** Summary of skin anti-aging drugs/ingredients studied in animal experiments.

No.	CAS No.	Compound Category	Classification of Compound Structures	Drugs/ Component	Animals	Modeling Methods	Modeling Time	Administration	Mechanisms	Reference
Oral administration (dietary)										
1	28448-85-3	Natural compounds	Flavonoids	Bavachalcone	WT and TET3+/− C57BL/6J mice	Intraperitoneal injection of paraquat 10 mg/kg	from w 6 to w 40	20 mg/kg by gavage every day	MDA↓, SOD↑, CAT↑, Hyp↑	Meng, 2019 [77]; Yuan, 2019 [79]
2		Natural compounds	Polyphenols	BPP	SD Rat	Subcutaneous injection of d-galactose 300 mg/kg	7 w	120 mg/kg by gavage for 7 w	MMP-1↓, collagen↑	Kai, 2010 [80]; Yu, 2020 [81]
3	458-37-7	Natural compounds	Polyphenols	Curcumin	BALB/c-nu/nu mice	Subcutaneous injection of d-galactose 125 mg/kg	3 w	80 mg/kg by gavage for 3 w	MDA↓, SOD↑, Hyp↑	Ben Yehuda Greenwald, 2017 [82]; Heng, 2010 [83]; Hui, 2016 [84]; Min, 2023 [85]
4	4852-22-6	Natural compounds	Polyphenols	Procyanidins	Kunming female mice	5% D-galactose 10 mL/kg was injected subcutaneously every day, and radiation was carried out the next day	40 d	50–100 mg/kg procyanidins orally administered for 30 d	MDA↓, SOD↑, GSH-Px↑, Hyp↑,	Weng, 2022 [86]; Juan, 2018 [87]; Fei, 2020 [88]; Rong, 2022 [89]
5	501-36-0	Natural compounds	Polyphenols	Resveratrol	Adult female Wistar rats	Excision of bilateral ovaries	31 d	Gavage 10, 30, and 60 mg/kg for 31 d	MDA↓, SOD↑, SIRT1 protein↑, Hyp↑	Leis, 2022 [90]; Dong,2015 [91]; Hecker, 2022 [92]
6	6640-24-0	Natural compounds	Polysaccharides	CPP	Kunming female Mice	Subcutaneous injection of 5% d-galactose solution 1 g/kg	42 d	50–150 mg/kg gavage for 32 days	MDA↓	Fude, 2023 [93]; Nong, 2014 [94]
7	107310-89-0	Natural compounds	Polysaccharides	LBP	Kunming female Mice	Subcutaneous injection of d-galactose 1000 mg/kg/d	42 d	5–20 mg kg^−1^ per day by gavage for 42 days	MDA↓, SOD↑, GSH-Px↑, Hyp↑	Zhu, 2022 [95]; Neves, 2021 [96]; Xia, 2015 [97]; Lei, 2023 [98]
8		Natural compounds	Polysaccharides	SMMP	Kunming female Mice	Subcutaneous injection of d-galactose 10 mL/kg/d +40 min, 5% UVB per day	40 d	Gavage 4 g/kg/d, starting from day 11 to day40	MDA↓, SOD↑, GSH-Px↑, Hyp↑	Nan, 2019 [99]; Hong, 2020 [100]
9	72909-34-3	Natural compounds	Quinones	PQQ	Bmi-1-knock out BKO mice	Knock out Bmi-1 gene		PQQ 4 mg per kilogram of feed for 4 w	Oxygen free radicals↓, collagen↑	Li, 2022 [101]; Yuan, 2017 [102]
Topical administration or injection										
10	78-70-6	Natural compounds	Monoterpenes	l-linalool	Female ICR mice	Subcutaneous injection of d-galactose 1.5 mg/g/d	30 d	Applied 5.0% to dorsal skin tissue for 30 d	Oxygen free radicals↓, MDA↓, SOD↑, Hyp↑	Keng, 2021 [104]
11	1094-61-7	Natural compounds	Nucleosides	NMN	ICR mice	12 h of UVB irradiation light/dark cycle per day	1 w	300 mg/kg by intraperitoneal injection for 3 w	SOD↑, TNF- α↓, IL-1 β↓, IL-6↓	Feng, 2022 [105]; Zhou, 2021 [106]
12	117-39-5	Natural compounds	Polyphenols	Quercetin	Male Kunming mice	Subcutaneous injection of d-galactose 200 mg/kg/d	8 w	Subcutaneous injection of 10–20 mg/kg/day for 8 w	SOD↑, T-AOC↑, GSH-Px↑, collagen↑	Shin, 2019 [107]; Xiao, 2022 [108]
13	10191-41-0	Natural compounds	Polyphenols	Vitamin E	Pig skin homogenate from Hainan breeding pigs			Using 20–100μL of Vitamin E (≥98%) and avoiding light reaction for 1 h	SOD↑, CAT↑	Nachbar, 1995 [118]; Lei, 2013 [109]
14	9012-76-4	Natural compounds	Polysaccharides	Chitosan	SD Rat			Daily application of chitosan (M = 106) 1% solution for 30 days, applied to dorsal skin	Hyp↑, collagen↑	Kong, 2018 [110]; Xing, 2022 [111]; Chun, 2018 [112]
15	102607-24-9	Natural compounds	Polysaccharides	GLP	Kunming Mice	Subcutaneous injection of d-galactose 1000 mg/kg/d	6 w	Applied 0.5% ganoderma lucidum polysaccharide to skin tissue for 42 days	DPPH↓, MDA↓, SOD↑, Hyp↑	Hu, 2019 [113]; Ye, 2020 [114]
16		Natural compounds	Polysaccharides	WGP	Mice	Subcutaneous injection of d-galactose 500 mg/kg/d	6 w	5 mg/mL of winter melon polysaccharide was applied to dorsal skin tissue at a rate of 2 m per day for 6 w	Oxygen free radicals↓, MDA↓, SOD↑, CAT↑, tyrosinase↓	Yibei, 2020 [115]; Xiao-yan, 2018 [116];
17	79-14-1	Synthetic compounds	Hydroxy acids	Hydroxyacetic acid	Kunming mice	Subcutaneous injection of d-galactose 1 mg/g/d	12 w	Applied 50% and 70% hydroxyacetic acid once every two weeks, applied to dorsal skin	Collagen↑, elastic fibers↑	Tang, 2019 [75]; Xiao, 2019 [117]

↑ or ↓ indicates increased or decreased, respetively.

**Table 3:** Summary of skin anti-aging drugs/ingredients studied in cell culture experiments.

No.	CAS No.	Compound Category	Classification of Compound Structures	Drugs/ Compounds	Cells	Modeling Methods	Modeling Time	Treatment	Mechanisms	References
1	29946-61-0	Natural compounds	Alkaloids	GX-50	Primary human skin fibroblasts	Hydrogen peroxide, (50 μM)	Once every two days, a total of 4 times	100 μM once every two days, a total of 4 times	ROS↓, MDA↓, the positive rate of β-Galactosidase↓	Ping, 2015 [119]; Shi, 2016 [141]; Xue-feng, 2013 [120]
2	1124-11-4	Natural compounds	Alkaloids	Ligustrazine	Human skin fibroblasts	UVA (10 J /d)	5 d	Ligustrazine hydrochloride injection 2 mL: 40 mg for 5 days	ROS↓, MMP-1↓, MMP-3↓, p53↓, the positive rate of β-Galactosidase↓	Liu, 2022 [121]; Ling, 2015 [122]
3	104594-70-9	Natural compounds	Catechols	CAPE	Human Dermal Fibroblasts-adult	UVA94.5% + UVB5%	48 h	5 uM for 48 h	MMP-1↓, collagen↑	Shin, 2019 [124]
4		Natural compounds	Flavonoids	Total flavonoids of Eurasian Convolvulus	Mouse skin fibroblasts	AGEs (0.1–0.2 g/L)	48 h	0.1 g/L–0.4 g/L Continuous action for 6 h	AGEs↓	Lu, 2018 [125]
5	110642-44-9	Natural compounds	Flavonoids	Epimedin C	HaCat cells	AGEs (1000 μg/mL)	1 h	100–300 μM for 24 h	ROS↓, MMPs↓, Cox-2↓, IL-6↓, IL-8↓	Bowen, 2019 [127]
6	61281-38-7, 61281-37-6, 61301-33-5	Natural compounds	Lignans	Schisandrin A, Schisandrin B, and Schisandrin C	HaCat cells	Hydrogen peroxide (50, 100, and 200 μM)	4 h	100 and 200 μM Schisandrin A, Schisandrin B, and Schisandrin C for 12 h	Oxidative damage↓	Lam, 2011 [128]; Wei, 2013 [129]
7	73-03-0	Natural compounds	Nucleosides	Cordycepin	Dermal fibroblasts	UVB	24 h	50–100μM for 24 h	MMPs↓	Lee, 2009 [130]
8	84687-43-4	Natural compounds	Saponins	Astragaloside IV	Aging human skin fibroblasts			5–20μg/mL administration monitoring within 24–120 h	Collagen↑	Chen, 2015 [131]; Xi, 2006 [132]
9	22427-39-0	Natural compounds	Saponins	Ginsenoside Rb1	Human dermal fibroblasts	600μM Hydrogen peroxide	3 h	500 μM for 2 h	ROS↓	Oh, 2015 [133]; Zhiwen, [134]; LIU Chun Xian, 2022 [135]
10	67-97-0	Natural compounds	Steroids	Vitamin D3	Human epidermal keratinocytes	UVB (50 mJ/cm^2^)	24 h	100 nM for 24 h	ROS↓, SOD↑	Bocheva, 2021 [137]; Chaiprasongsuk, 2019 [138]
11	657-24-9	Synthetic compounds	Biguanides	Metformin	Human skin fibroblasts	UVA 5 J/cm^2^ for consecutive 3 days	24 h	1 mmol/L for 72 h	ROS↓, SOD↑, MMP-1↓, MMP-3↓, the positive rate of β-Galactosidase↓	Chen, 2022 [139]; Yi, 2017 [140]

↑ or ↓ indicates increased or decreased.

## Data Availability

The data presented in this study are available in the article.
